# Overcoming mTOR Inhibitor Resistance: From Biological Basis to Therapeutic Strategies

**DOI:** 10.3390/biomedicines14081732

**Published:** 2026-07-31

**Authors:** Xi-Feng Jin, Ling Liu, Jun-Jun Zhang

**Affiliations:** Departments of Gastroenterology, The Second Affiliated Hospital of Zhejiang University School of Medicine, Hangzhou 310009, China; llee2022@zju.edu.cn (L.L.); jjzhangee2022@zju.edu.cn (J.-J.Z.)

**Keywords:** mTOR inhibitor resistance, autophagic adaptation, immune evasion, metabolic reprogramming, tumor heterogeneity, m6A methylation, non-coding RNAs, multiomics

## Abstract

Resistance to mTOR inhibition is mediated by multiple adaptive mechanisms, including feedback activation of the PI3K/AKT/mTOR pathway, autophagic adaptation, metabolic reprogramming, immune evasion, tumor heterogeneity, and microenvironmental crosstalk. Recent research has also elucidated the crucial role of RNA modifications, particularly m6A methylation, and the regulatory impact of non-coding RNAs in facilitating tumor adaptation to mTOR blockade. Accordingly, novel therapeutic approaches are emerging. The combination of PI3K/AKT/mTOR inhibitors with immune checkpoint blockades represents a promising strategy for overcoming therapeutic resistance. Additionally, next-generation mTOR inhibitors and synthetic lethality strategies are being tailored to target specific tumor profiles. Furthermore, advancements in multi-omics technologies and AI-powered predictive models are transforming personalized cancer treatment. This comprehensive review delves into the molecular intricacies of mTOR inhibitor resistance and explores innovative strategies aimed at enhancing therapeutic outcomes and improving patient responses.

## 1. Introduction

The mechanistic target of rapamycin (mTOR) pathway serves as a pivotal regulator of cellular functions, governing essential processes such as protein synthesis, autophagy, and metabolism. As a central hub for maintaining cellular homeostasis, mTOR integrates diverse signals from nutrients, growth factors, and cellular energy levels [1,2]. However, in cancer, mTOR signaling is frequently dysregulated, resulting in uncontrolled tumor growth, hyper-proliferation, and therapeutic resistance. mTOR inhibitors, particularly rapalogs such as everolimus and temsirolimus, have attracted significant clinical interest for treating various cancers, including renal cell carcinoma, breast cancer, and neuroendocrine tumors (NETs). Initial phase III trials demonstrated that everolimus substantially improved progression-free survival compared with control regimens in both advanced renal cell carcinoma and hormone receptor-positive breast cancer [3]. Despite these promising outcomes, further analyses revealed elevated rates of disease progression following prior therapy in the trials BOLERO-3, RECORD-3, and EVOLVE-1, thereby posing significant challenges in the long-term management of cancer patients [4,5,6].

**Figure 1:**
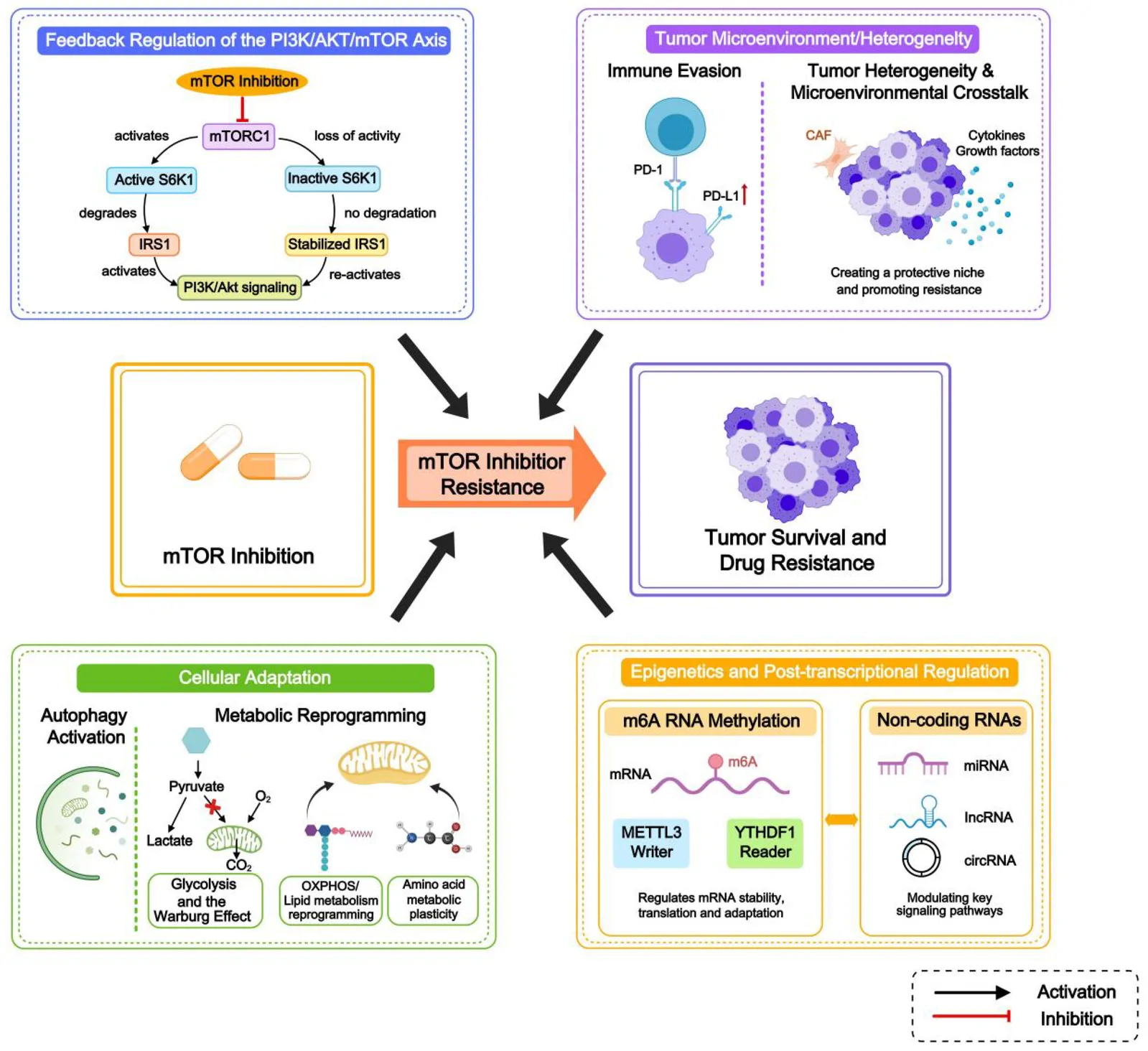
Integrated mechanisms of resistance to mTOR inhibitors in human cancers.

This review investigate the intricate molecular mechanisms underlying resistance to mTOR inhibitors, focusing on pivotal factors such as feedback regulation, metabolic reprogramming and tumor–microenvironment crosstalk. Moreover, it highlights innovative therapeutic strategies designed to overcome these resistance mechanisms. Recent advances in multi-omics technologies—encompassing genomics, transcriptomics, proteomics, and metabolomics—have substantially deepened our understanding of the heterogeneous landscapes that underpin acquired and intrinsic resistance. Notably, single-cell and spatial multi-omics approaches are increasingly used to resolve clonal dynamics and microenvironmental adaptations, which bulk analyses often miss. Concurrently, the integration of artificial intelligence (AI) into cancer biology is catalyzing the transition from descriptive to predictive and prescriptive practices within the field of oncology. Machine learning models, particularly when applied to large-scale multi-omics and longitudinal clinical data, are increasingly utilized to identify novel resistance biomarkers, stratify patient responses, and recommend therapeutic regimens. These AI-driven frameworks are pivotal for designing next-generation combination therapies (e.g., co-targeting mTOR with parallel adaptive pathways or metabolic checkpoints) and for advancing precision medicine strategies that circumvent or overcome resistance to current mTOR inhibitors. In summary, this review synthesizes how the convergence of high-resolution multi-omics and AI-driven analytics is transforming our understanding of resistance mechanisms to mTOR inhibitors and informing the strategy-drive development of personalized, adaptive treatment paradigms (Figure 1, Table 1) [7].

## 2. Mechanisms Underlying Resistance to mTOR Inhibitors via Feedback Loops and Bypass Pathway Activation of the PI3K/AKT/mTOR Axis

The clinical utility of mTOR inhibitors is often limited by the emergence of resistance, which may be intrinsic or acquired after prolonged drug exposure [76]. Resistance to mTOR-targeted drugs is a complex, multifactorial process driven by dynamic adaptive mechanisms within tumor cells. This process undermines the therapeutic potential of this drug class and constitutes a significant unmet clinical need [77].

### 2.1. Positive Feedback Loop of IRS-1/PI3K Induced by mTORC1 Inhibition

Inhibition of the mechanistic target of rapamycin complex 1 (*mTORC1*) disrupts a key negative feedback loop, thereby causing the upregulation and stabilization of insulin receptor substrate-1 (IRS-1). This results in enhanced activation of the upstream phosphatidyl-inositol-3-kinase (*PI3K*) signaling, which paradoxically reactivates the same pathway that the mTOR inhibitors aim to suppress. This feedback activation constitutes a primary mechanism of resistance by sustaining pro-survival signals [8]. And this compensatory signaling highlights the inherent challenge of targeting a single node within a highly interconnected network (Figure 1, Table 1).

### 2.2. Sustained AKT Activation Drives Maintenance of Survival Signals

This persistent *AKT* activity can be driven through multiple mechanisms, including the loss of negative regulators such as phosphatase and tensin homolog (*PTEN*) or direct upstream activation. Studies have demonstrated that in rapamycin-resistant cancer cells, the upregulation of Raptor and the activation of *AKT* signaling are associated with poor therapeutic response [9]. The sustained activation of *AKT* promotes cell survival and proliferation, directly counteracting the cytotoxic effect of mTOR inhibition and thereby driving resistance. Furthermore, overexpression of positive regulators like miR-181c can enhance this Raptor/Akt signaling axis, and thus contribute to mTOR resistance [9]. In prostate cancer, overexpression of tumor protein D52 (TPD52) induces resistance to everolimus and rapamycin. This occurs via an mTOR-independent mechanism that involves hyperactivating the PI3K/AKT pathway and maintaining high levels of phosphorylated (inactive) 4E-BP1 (eukaryotic translation initiation factor 4E-binding protein 1) [10] (Figure 1, Table 1).

### 2.3. Upregulation of Alternative Survival Pathways and Activation of Bypass Signaling

Tumor cells evade targeted therapy by upregulating compensatory signaling pathways. When mTOR is inhibited, for instance, cells often increase expression of receptor tyrosine kinases (*RTKs*) such as insulin-like growth factor receptor (*IGF-1R*) and human epidermal growth factor receptor 2 (*HER2*), which activate parallel or downstream cascades (e.g., PI3K/AKT and MAPK) to circumvent this blockade. In clear cell renal cell carcinoma (ccRCC), resistance to mTOR inhibitors has been linked to feedback activation of multiple RTKs and their effectors, including extracellular signal-regulated kinase (*ERK*) and SRC proto-oncogene [11]. Conversely, in other treatment settings, such as thyroid cancer cells resistant to RET inhibitors, the AKT-mTOR pathway mediates a critical compensatory survival mechanism [12], thereby underscoring the bidirectional crosstalk between distinct signaling pathways. In breast cancer, eukaryotic elongation factor-2 kinase *(eEF-2K*) mediates cytoprotective autophagy and negative-feedback activation of Akt in response to mTOR inhibitors [13]. Additionally, activation of growth-associated transcription factors such as activating protein-1 (AP-1) has been observed in resistant cells [9]. In hepatocellular carcinoma, the E2F transcription factor 7 (E2F7) promotes cancer cell resistance to sirolimus by suppressing mTORC1 via TSC1 and by stabilizing HIF-1α, which in turn activates its downstream target genes [9] (Figure 1, Table 1).

## 3. Autophagy-Mediated Resistance to mTOR Inhibitors

### 3.1. Autophagy as a Survival Mechanism to Clear Damaged Organelles and Provide Energy

mTORC1 is a potent negative regulator of autophagy, primarily by phosphorylating and inhibiting the autophagy-activating UNC-51-like kinase 1 (*ULK1*) complex. Pharmacological inhibition of mTORC1 relieves this suppression, thereby promoting the dephosphorylation and activation of ULK1, which initiates autophagosome formation. In glioblastoma, PI3K/AKT/mTOR inhibitors activate cytoprotective autophagy, which can either sensitize cells to therapy or promote survival under drug pressure depending on the specific cellular context [14].

Autophagy therefore represents a double-edged sword in mTOR inhibitor-based cancer therapy. On one hand, it functions as a pro-survival mechanism by clearing the damaged components and recycling the macromolecules to provide energy and metabolic substrates, allowing cancer cells to tolerate mTOR inhibition-induced metabolic stress [15]. On the other hand, such adaptive responses enable tumors to enter dormancy or resume proliferation, thereby contributing to acquired resistance (Figure 1, Table 1).

### 3.2. Combination Therapy of Autophagy Inhibitors and mTOR Inhibitors

Given the protective role of autophagy in mediating resistance, combining mTOR inhibitors with autophagy inhibitors is a promising strategy to enhance cytotoxicity. Preclinical studies across diverse cancer types support this therapeutic strategy. In models of breast cancer, the combination of the mTOR inhibitor rapamycin and the autophagy inhibitor chloroquine demonstrated a synergistic effect [13]. Similarly, in lung carcinoid models, the autophagy inhibitor chloroquine potentiated the anti-tumor effects of mTOR inhibitors such as everolimus [16] (Figure 1, Table 1).

## 4. Metabolic Plasticity in mTOR Therapeutic Escape

Metabolic reprogramming is a hallmark of cancer that enables cells to sustain proliferation under nutrient-limited conditions and therapeutic stress. The mTOR pathway is a central regulator of cellular metabolism, controlling glycolysis, lipid metabolism, nucleotide synthesis, and mitochondrial function [17]. Upon mTOR inhibition, cancer cells undergo adaptive metabolic reprogramming, thereby promoting survival and driving the emergence of therapeutic resistance [18] (Figure 1, Table 1).

### 4.1. The Role of Glycolysis and the Warburg Effect in Mediating Resistance to mTOR Inhibitors

mTOR-resistant tumors maintain high glycolytic flux despite mTOR blockade, a metabolic phenotype characteristic of the Warburg effect. Quantitative metabolic analyses have demonstrated that these tumors produce significantly more lactate even in the presence of functional mitochondria [19,20]. This glycolytic persistence is primarily driven by mTORC1-dependent hypoxia-inducible factor-1α (HIF-1α) stabilization, which transcriptionally upregulates key glycolytic enzymes, including hexokinase 2 (HK2) and pyruvate kinase M2 (PKM2). Notably, even under mTOR inhibition, AMPKα2-mediated phosphorylation of 6-phosphofructo-2-kinase/fructose-2,6-bisphosphatase 3 (PFKFB3)—a potent allosteric activator of glycolysis—maintains glycolytic flux and promotes tumor cell proliferation [21,22,23].

Clinical imaging using ^18^F-FDG PET consistently demonstrates increased glucose uptake in mTOR inhibitor-resistant lesions, a finding that correlates with AKT3-dependent trafficking of glucose transporter type 1 (GLUT1) to the plasma membrane [10]. This observation is further supported by mass spectrometry-based proteomic analyses, which confirmed a marked elevation of surface GLUT1 levels in resistant tumors [48]. Functional genomics studies have identified co-amplification of MYC and PGC-1α in a subset of resistant cases, a genomic alteration that facilitates NADH shuttling between mitochondria and the cytosol, thereby supporting redox balance and sustained glycolysis [24]. Despite these mechanistic insights, the narrow therapeutic index of most glycolysis inhibitors remains a major clinical challenge. Therefore, the identification of predictive biomarkers for patient stratification is urgently needed to enable precision-targeted metabolic interventions.

### 4.2. Mitochondrial Metabolic Rewiring Drives Resistance to mTOR Inhibitors via Compensatory Bioenergetic Networks

Resistant tumors escape mTOR inhibition through metabolic reprogramming that favors oxidative phosphorylation (OXPHOS). Isotope tracing studies have shown that mTOR inhibitor-resistant tumors exhibit a 3.8-fold increase in the incorporation of [U-^13^C]glutamine into the tricarboxylic acid (TCA) cycle [24]. Suppression of mTORC1 relieves the translational control of PGC-1α (peroxisome proliferator-activated receptor gamma coactivator 1-alpha), a master regulator of mitochondrial biogenesis, thereby inducing cristae remodeling [25]. Concomitantly, AMPKα2-mediated phosphorylation of NRF1 (nuclear respiratory factor 1) upregulates electron transport chain (ETC) complexes while suppressing glycolysis [25]. Clinically, [^13^C]acetate PET imaging has confirmed increased mitochondrial oxidation in resistant tumors, along with elevated serum levels of mitochondrial DNA (mtDNA).

Therapeutic dual targeting of mitochondrial metabolism has emerged as a promising strategy. In preclinical models, the combination of the complex I inhibitor phenformin with everolimus substantially reduced glioblastoma tumor volume [25]. Similarly, the Drp1 inhibitor Mdivi-1 disrupted mitochondrial fission, leading to marked reduction in cristae fragmentation and sensitizing hepatocellular carcinomas (HCCs) to rapalogs [26]. A phase II trial evaluated the ETC complex I inhibitor IACS-010759 in combination with an mTOR inhibitor in patients with refractory melanoma, and a subset of participants achieved objective responses (per RECIST criteria). Metabolic flux analysis further confirmed on-target activity, evidenced by normalization of the lactate-to-pyruvate ratio [27]. Despite these encouraging results, the clinical development of mitochondrial inhibitors has been hindered by dose-limiting toxicities, such as lactic acidosis and peripheral neuropathy, thereby necessitating rigorous patient monitoring alongside tumor-specific delivery strategies.

### 4.3. Lipid-Metabolic Reprogramming Maintains mTOR Resistance via Membrane Structural Remodeling

The resistance of tumors to mTORC1 inhibition drives a metabolic shift towards increased lipid scavenging, as evidenced by upregulation of CD36-mediated palmitate uptake and of FABP4 expression in breast cancer models [28]. This metabolic shift is driven by sterol regulatory element-binding protein-2 (*SREBP2)* cleavage, which leads to LDL receptor overexpression and subsequent cholesterol uptake from lipoproteins in the tumor microenvironment (TME) [29]. These findings indicate that tumors compensate for mTORC1-mediated suppression of de novo lipogenesis by enhancing exogenous lipid internalization. However, the clinical relevance of these pathways has yet to be established [30].

Stearoyl-CoA desaturase 1 (*SCD1*) catalyzes the conversion of saturated fatty acids into monounsaturated fatty acids (MUFAs), thereby maintaining membrane fluidity. Inhibition of SCD1 reversed everolimus resistance in RCC by disrupting PI3K/AKT signaling in lipid rafts and triggering ferroptosis through peroxidation cascades [31]. Enhanced fatty acid oxidation (FAO) further contributes to mTOR inhibitor resistance, as evidenced by the markedly increased incorporation of [U-^13^C]-palmitate into TCA cycle intermediates in resistant tumors. The *CPT1* inhibitor ST1326 substantially reduced ATP production, increased reactive oxygen species (ROS), and induced significant tumor regression in prostate cancer PDX models [32]. A phase Ib trial evaluating the combination of a carnitine O-palmitoyltransferase 1 (CPT1) inhibitor with rapalogs in patients with refractory clear cell renal cell carcinoma (ccRCC) reported objective responses in a subset of patients, suggesting that circulating MUFA levels may serve as potential predictive biomarkers [33,34]. Although these data highlight the therapeutic promise of targeting lipid metabolism, tumor-type-specific lipid metabolic dependencies and the risk of compensatory metabolic rewiring pose substantial challenges to clinical translation.

### 4.4. Amino Acid Metabolic Plasticity Drives Escape from mTOR Inhibition Through Nitrogen-Carbon Coupling

Resistant tumors exhibit adaptive glutamine metabolism driven by the MYC/ATF4 axis. By using [U-^13^C]-glutamine tracing, researchers have observed a marked increase in TCA cycle flux and substantially enhanced nucleotide ^15^N incorporation [35]. Inhibition of mTORC1 triggers the ATF4-mediated upregulation of solute carrier family 1 member 5 (*SLC1A5*) and the stabilization of glutaminase 1 (*GLS1)*, thereby maintaining glutamate levels for glutathione synthesis. Concurrently, MYC amplification induces glutaminase-2(*GLS2*) overexpression, which mediates α-ketoglutarate(α-KG)-dependent histone demethylation, thereby preserving stemness in resistant clones [36]. Preclinical studies combining the GLS inhibitor CB-839 with everolimus resulted in a significant reduction in xenograft volume via the inhibition of both mTORC1 signaling and NRF2-mediated antioxidant pathways [37]. However, due to the functional redundancy in amino acid metabolism and the potential for rapid adaptive rewiring, single-agent therapies may demonstrate limited long-term efficacy, highlighting the need for combination strategies.

### 4.5. Cuproptosis and mTOR-Inhibitor-Mediated Therapeutic Escape

Cuproptosis, a newly characterized form of copper-dependent programmed cell death, has emerged as a potential vulnerability in mTOR inhibitor-resistant tumors [72]. Copper is essential for mitochondrial enzymes such as cytochrome c oxidase; however, excessive copper accumulation targets lipoylated TCA cycle proteins, triggering proteotoxic stress and cell death [73]. Single-cell RNA sequencing has reveals that specific subsets of mTOR-inhibited cancer cells with elevated mitochondrial activity are more susceptible to copper-induced cell death [74]. Proteomic analyses further demonstrate that cancer cells exhibit alterations in lipoylated mitochondrial proteins, thereby conferring hypersensitivity to copper ionophores, which promote copper accumulation and eradicate resistant cell populations in preclinical models [75]. Despite this mechanistic rationale, the field is still in its infancy. Key challenges include optimizing the specificity of copper ionophores, identifying predictive biomarkers for patient selection, and mitigating the risk of systemic copper toxicity. Further studies are needed to determine whether cuproptosis-directed strategies can be safely combined with mTOR inhibitors.

## 5. Tumor Heterogeneity and mTOR Inhibitor Resistance

Intratumoral heterogeneity is a complex phenomenon driven by genetic mutations, epigenetic changes, and variable gene expression within the tumor. Recent advancements in single-cell RNA sequencing technology have shed light on the diverse cellular makeup of tumors, revealing distinct subpopulations of cancer cells with unique metabolic profiles, pathway activations, and responses to treatment. These findings have provided a more nuanced insight into tumor dynamics and the development of personalized treatment strategies specific to the tumor subtypes [41] (Figure 1, Table 1).

### 5.1. Genetic Heterogeneity and mTOR Inhibitor Resistance

Research utilizing single-cell RNA sequencing (scRNA-seq) has revealed significant heterogeneity in metabolic functions, signaling pathways and responses to treatment among subpopulations of cancer cells. A recent study of a large cohort of resistant tumors elucidated the diverse mechanisms driving resistance to mTOR inhibitors. The analysis identified three distinct evolutionary trajectories. Notably, a substantial proportion of tumors acquired a specific hotspot mutation in the *PIK3CA* gene, which led to a marked increase in *pAKT* signaling. The BOLERO-4 trial demonstrated that intermittent treatment with gedatolisib reduced intratumoral heterogeneity. This reduction was associated with a substantially prolonged median progression-free survival (PFS) compared with everolimus, while maintaining a manageable toxicity profile [38,47]. Such pre-existing variants can be selectively enriched under therapeutic pressure, which in turn drives rapid treatment failure.

### 5.2. Clonal Selection and Evolutionary Dynamics Under Therapeutic Pressure

The selective pressure imposed by mTOR inhibitors drives the Darwinian evolution of tumor cell populations. Sensitive clones are eliminated, while pre-existing resistant subclones or de novo resistant variants proliferate. This dynamic process of clonal selection results in the emergence of a resistant tumor phenotype. Research into renal clear cell carcinoma has elucidated the emergence of resistance mechanisms, thereby undermining the efficacy of mTOR inhibitors and prompting the development of therapeutic strategies to circumvent this adaptive response [39]. Therapeutic pressure progressively remodels the tumor ecosystem, resulting in a more aggressive and treatment-refractory disease state.

### 5.3. Role of Cancer Stem Cells (CSCs) in mTOR Inhibitor Resistance

Cancer stem cells (CSCs) constitute a subpopulation characterized by self-renewal capacity and inherent resistance mechanisms, including enhanced DNA repair, drug efflux, and metabolic plasticity. They are widely implicated in tumor recurrence after therapy. Some PI3K/mTOR inhibitor-resistant cancer cells exhibit elevated expression of markers typical of cancer stem cells (CSCs) [40]. These CSCs can persist despite mTOR inhibitor treatment and subsequently drive tumor recurrence, thus being established as crucial therapeutic targets.

## 6. The Role of Epigenetics and Post-Transcriptional Regulation in Drug Resistance

### 6.1. Targeting m6A Regulation as a Novel Strategy for Overcoming mTOR Inhibitor Resistance

Given the central role of m^6^A in regulating gene expression and its dysregulation in cancer, modulating the m^6^A modification machinery represents a promising therapeutic avenue for overcoming drug resistance. m6A dynamically regulates genes in pathways such as the PI3K/AKT/mTOR axis; therefore, targeting m6A regulators may resensitize therapy-resistant cancer cells [78]. Researchers have utilized the expression patterns of m6A regulators—including methyltransferase-like 3 *(METTL3*), obesity-associated protein (*FTO*), and YTH m6A RNA-binding protein 1 (*YTHDF1)*—to construct prognostic models for gastrointestinal cancers [48] (Figure 1, Table 1).

Three distinct m6A modification patterns associated with differential responses to mTOR inhibitors were identified in tumor samples [49]. Studies have demonstrated that METTL3 upregulates GLUT1 expression, thereby accelerating tumor progression and leading to resistance to mTOR inhibitors. Consistently, preclinical evidence demonstrates that METTL3 inhibition synergizes with mTOR inhibitors by disrupting hypoxia-inducible factor-2α (*HIF-2α*)-driven metabolic adaptation [50,51]. ALKB homolog 5 (*ALKBH5*), an m6A demethylase, confers resistance to mTOR inhibitors by inducing mTORC2 addiction through the stabilization of rapamycin-insensitive companion of mTOR (*RICTOR*) mRNA and consequent enhancement of 4EBP1 phosphorylation. In high-ALKBH5 triple-negative breast cancer (TNBC) models, ALKBH5 drives tumor glycolysis, a process effectively suppressed by the ALKBH5 inhibitor W23-1006 [52]. Beyond m6A, the m^6^A reader YTHDF1 promotes glycolysis by recognizing m^6^A-modified GLUT1 transcripts. Furthermore, CRISPR screening has revealed synthetic lethality between YTHDF1 and pyruvate kinase M2 (*PKM2)* in hepatocellular carcinoma (HCC) models [53]. While these synthetic lethal interactions present promising therapeutic avenues, they have yet to be validated in vivo and in patient-derived models. Beyond m^6^A, chemical mapping approaches have identified m^1^A and m^5^C modifications as additional regulators of the mTOR network [54,55]. Despite this encouraging preclinical evidence, the clinical translation of epitranscriptomic targeting remains at an early stage. Key challenges include ensuring adequate penetration into tumor tissues, minimizing potential off-target effects on the normal epitranscriptome, and overcoming redundancy within the RNA modification machinery.

### 6.2. Non-Coding RNAs and mTOR Inhibitor Resistance

Non-coding RNAs (ncRNAs), including microRNAs (miRNAs) and long non-coding RNAs (lncRNAs), have emerged as critical regulators of mTOR signaling and mediators of resistance to mTOR inhibitors. These ncRNAs regulate the key pathway components, thereby allowing cancer cells to circumvent therapeutic blockade.

#### 6.2.1. MicroRNA Networks in mTOR Inhibitor Therapeutic Escape

Multiple miRNAs have been implicated in regulating mTOR signaling flexibility. For instance, miR-214 directly inhibits *TSC2*, leading to *mTORC1* activation and promoting cell survival under stress conditions [56]. The combination of miR-4634 and RAD001 demonstrated synergistic antitumor effects by inhibiting cell proliferation, migration, and colony formation. Co-treatment with miR-4634 and RAD001 represents a potential mTOR-targeted therapeutic strategy for NSCLC patients [57]. The miR-17-5p/PTEN/AKT axis functions via a dual feedback mechanism, whereby decreased miR-17-5p leads to upregulated PTEN expression and diminished AKT phosphorylation. Analysis of data from The Cancer Genome Atlas (TCGA) revealed that elevated miR-21 expression correlates with reduced TSC2 levels and diminished rapamycin sensitivity [58].

Therapeutic restoration of tumor-suppressive miRNAs has shown promise in preclinical studies and early clinical trials. For example, delivery of miR-34a via lipid nanoparticles markedly reduced pS6K levels in prostate xenograft models, and a phase I trial subsequently reported objective responses in a subset of patients with mTOR inhibitor-resistant cancers [59,60]. However, these data remain preliminary, as the findings are correlational and derived from bulk tumor analyses, thereby precluding any causal inference. Furthermore, functional validation using appropriate in vivo models has yet to be performed. In addition, the safety profile of miRNA-based therapeutics—particularly off-target effects—warrants further evaluation in larger cohorts.

#### 6.2.2. Long Non-Coding RNAs Mediate mTOR Inhibitor Resistance

Long non-coding RNAs (lncRNAs) have emerged as critical regulators of mTOR signaling and therapy resistance. In hepatocellular carcinoma (HCC), lncRNA H19 stabilizes mTORC1 complexes and promotes epithelial–mesenchymal transition (EMT) through interacting with Raptor and enhancing Twist-related protein 1 (Twist1) expression, thereby sustaining mTOR activity. In breast cancer patients, elevated H19 levels are associated with shorter progression-free survival (PFS) following everolimus treatment. However, given the retrospective nature of these findings, prospective validation is essential to establish H19 as a reliable predictive biomarker [61]. Beyond H19, metastasis-associated lung adenocarcinoma transcript 1 *(MALAT1*) represents another lncRNA that confers mTOR resistance via distinct mechanisms. *MALAT1* modulates the tumor microenvironment by secreting miR-21-enriched exosomes, thereby reprogramming cancer-associated fibroblasts and maintaining AKT signaling in drug-resistant tumors [62]. Preclinical studies have demonstrated that targeting *MALAT1* with locked nucleic acid antisense oligonucleotides (ASOs)—either alone or in combination with rapamycin—significantly inhibits tumor growth in patient-derived xenograft models and reduces metastatic burden in lung cancer models, highlighting the critical role of lncRNA-driven mTOR resistance [63,64]. Despite the therapeutic potential of ASO-based strategies for interfering with these signaling axes, their clinical translation remains hindered by low delivery efficiency, poor tissue penetration, and off-target effects. Emerging nanotechnology platforms, such as pH-sensitive lipid nanoparticles, have demonstrated improved tumor accumulation and reduced hepatotoxicity, although they remain at an early stage of development [64].

#### 6.2.3. ceRNA Networks in mTOR Inhibitor-Mediated Therapeutic Escape

Competing endogenous RNA (ceRNA) networks constitute an additional layer of regulatory complexity, as exemplified by the circHIPK3/miR-381-3p axis. circHIPK3 functions as a sponge by sequestering miR-30a-3p, resulting in increased AKT phosphorylation at a specific serine residue and thereby promoting escape from mTOR inhibition [65]. A phase II trial demonstrated that circHIPK3 antisense oligonucleotide (ASO) therapy significantly reduced the incidence of hepatic metastases in colorectal cancer patients with elevated plasma levels of this circRNA [66]. Furthermore, combining this ASO approach with miR-326 restoration using lipid nanoparticles substantially improved treatment efficacy in patient-derived xenograft (PDX) models [67]. Nevertheless, the clinical applicability of ceRNA-targeting strategies is hindered by significant challenges: the need for optimized delivery vectors, the absence of reliable biomarkers for patient selection, and the incomplete understanding of network redundancy [68,69].

## 7. Microenvironmental and Immunological Mechanisms Underlying Escape from mTOR-Targeted Therapy

### 7.1. Microenvironmental Drivers’ Therapeutic Resistance to mTOR Inhibitors

Emerging evidence from spatial omics has delineated three distinct tumor microenvironment (TME) resistance patterns that collectively contribute to therapeutic escape from mTOR inhibitors. The first pattern, driven by hypoxia, is predominantly observed in the tumor core, characterized by elevated levels of HIF-1α and vascular endothelial growth factor-A (*VEGFA*). The second pattern of resistance, known as stroma-mediated resistance, is typically observed at the tumors periphery and is associated with FAP+ cancer-associated fibroblasts and increased levels of collagen type I alpha 1 chain (*COL1A1*). The third resistance pattern, characterized by an immune-excluded phenotype, is defined by the spatial exclusion of CD8+ T cells. Based on these findings, tailored treatment strategies have been proposed. For tumors exhibiting the hypoxia-driven resistance pattern, HIF-2α inhibitors such as belzutifan may be effective. Stroma-mediated resistance may be targeted with focal adhesion kinase (*FAK*) inhibitors such as defactinib, whereas tumors displaying the immune-excluded phenotype may benefit from C-X-C chemokine receptor type 4 (*CXCR4*) antagonists like plerixafor [70,71] (Figure 1, Table 1).

### 7.2. Dual Modulatory Effect of mTOR Inhibitors on the Immune System

Recent single-cell analyses have revealed that mTOR inhibitors exert a context-dependent, dual modulatory effect on the immune system. On the one hand, these inhibitors impair regulatory T cell (Treg) differentiation by reducing Forkhead box P3 *(FoxP3*) expression, thus relieving Treg-mediated suppression of CD8+ effector T cells [41]. On the other hand, they enhance the stemness of CD8+ tumor-infiltrating lymphocytes (TILs) via metabolic reprogramming, thereby fostering a PD-1+TCF1+ progenitor T cell state. mTOR inhibition has been shown to enhance antitumor immunity in multiple tumor models. In melanoma, intermittent mTOR inhibition expands the progenitor T cell population, and its combination with anti-PD-1 therapy achieves a 62% complete response rate, suggesting that this csynerogisstic interaction can reverse resistance [42]. In non-small-cell lung cancer (NSCLC), temsirolimus reprograms M2-polarized tumor-associated macrophages (TAMs) into an M1-like pro-inflammatory phenotype, enhancing anti-PD-1 efficacy [24]. However, this beneficial effect is often counterbalanced by compensatory crosstalk, characterized by increased Treg infiltration and upregulation of indoleamine 2,3-dioxygenase 1 (IDO1) in tumor cells—both of which suppress CD8^+^ T cell activity [44,45]. Furthermore, in the KEYNOTE-427B trial of PD-L1-positive triple-negative breast cancer, the addition of everolimus to pembrolizumab enhanced the objective response rate and prolonged the median duration of response, confirming that mTOR inhibition can potentiate checkpoint blockade [46]. Conversely, detrimental effects of mTOR inhibition have also been reported. In pancreatic adenocarcinoma, sustained mTOR blockade activates cancer-associated fibroblasts (CAFs), which secrete C-X-C motif ligand 12 (CXCL12) and recruit immunosuppressive cells, thereby shifting the balance toward resistance [43]. In renal cell carcinoma, everolimus-induced Treg expansion can be reversed by CCR4 inhibition, thus highlighting a therapeutically targetable node within this crosstalk network [44]. These findings highlight that the balance between antitumor immunity and therapeutic efficacy—which ultimately modulates the potential to overcome mTOR inhibitor resistance and depends on tumor types, dosing schedules, and the baseline state of immune crosstalk.

## 8. Application of Artificial Intelligence and Machine Learning in Research on mTOR Inhibitor Resistance

### 8.1. Artificial Intelligence for High-Dimensional Integration of Multi-Omics Data and Pattern Recognition

Multi-omics data derived from resistant tumors are typically high-dimensional and heterogeneous, posing substantial challenges for traditional statistical methods. Artificial intelligence (AI) and machine learning (ML) provide a powerful solution by integrating genomic, transcriptomic, and proteomic datasets to uncover resistance-associated patterns. For instance, upregulation of Raptor or activation of cytoprotective autophagy frequently occurs across multiple cancer types in the context of mTOR inhibitor resistance. However, such signals can be subtle or synergistic, making them difficult to detect reliably using any single omics layer or conventional univariate analysis. In contrast, AI/ML models excel at identifying these subtle yet biologically meaningful combinatorial signatures, thereby revealing novel resistance mechanisms [9,14]. Importantly, the field is increasingly shifting toward interpretable AI approaches that not only predict resistance but also pinpoint testable biological drivers, thus bridging the gap between computational pattern recognition and mechanistic understanding.

### 8.2. Prediction of mTOR Inhibitor Response Based on Patient Genomic Data

Machine learning models are increasingly being developed to predict patient responses to mTOR inhibitors based on genomic profiles, aiming to personalize therapy and overcome resistance. Specific genomic alterations—such as mutations in PIK3CA or PTEN, and overexpression of genes like TPD52 or E2F transcription factor 7 (E2F7)—have been linked to mTOR inhibitor resistance in multiple malignancies, including breast cancer, prostate cancer, and hepatocellular carcinoma [10]. Based on these features, these predictive models can distinguish responders from non-responders. Specifically, a model using cellular morphometric biomarkers from biopsy images accurately predicted the resistance to androgen deprivation therapy in patients with prostate cancer. Such resistance was associated with mTOR pathway activity, suggesting that non-responders to androgen deprivation therapy might instead benefit from mTOR inhibitors [79].

### 8.3. Digital Pathology-Based Analysis to Predict Therapeutic Efficacy

Artificial intelligence-powered digital pathology enables the prediction of therapeutic efficacy and resistance by extracting quantitative features of the tumor microenvironment (TME) from histopathological images [80,81]. In clear cell renal cell carcinoma, tumor-derived exosomes can promote resistance to mTOR inhibitors by modulating signaling pathways within the TME [82]. AI models using whole-slide images can also quantify TME characteristics, such as immune cell infiltration and stromal composition. For example, a radiomics model based on magnetic resonance imaging (MRI) data was developed to non-invasively predict ribosomal protein S6 kinase (S6K) expression in hepatocellular carcinoma. This capability facilitates clinical decision-making regarding mTOR inhibitor therapy [83]. Thus, by correlating TME features with molecular resistance mechanisms, digital pathology offers a non-invasive strategy for prognostic stratification and optimization of treatment planning.

## 9. Emerging Therapeutic Strategies and Future Directions to Overcome mTOR Inhibitor Resistance

### 9.1. Combination of mTOR Inhibitors with PI3K/AKT Inhibitors

Targeting upstream nodes of the PI3K/AKT/mTOR pathway, such as *PI3K* or *AKT*, in combination with mTOR inhibitors constitutes a vertical inhibition strategy to overcome feedback activation and acquired resistance. The dual PI3K/mTOR inhibitor NVP-BEZ235 (BEZ235) has demonstrated efficacy in overcoming resistance across multiple models. For instance, in doxorubicin-resistant leukemia cells, BEZ235 reduces cell viability, induces G0/G1 arrest, and promotes apoptosis by downregulating the PI3K/AKT/mTOR signaling axis and its downstream effectors [84]. Similarly, studies in bladder cancer cells indicate that combining the AKT inhibitor AZD5363 with the mTOR inhibitors AZD2014 or BEZ235 produces synergistic anti-proliferative effects, suppressing PI3K/AKT/mTOR signaling more effectively than either agent alone [85]. This vertical combination strategy is also being explored in other cancer types, further highlighting the potential of co-targeting PI3K/AKT and mTOR to circumvent resistance [86].

### 9.2. Combination of mTOR Inhibitors with Epigenetic Drugs or Metabolic Inhibitors

Epigenetic modulation has been shown to enhance the efficacy of mTOR inhibitors. In endometrial cancer, inhibition of Lysine-specific histone demethylase 1A (KDM1A/LSD1) synergizes with rapamycin by attenuating mTORC2 complex gene expression and preventing feedback activation of AKT, thereby reducing tumor growth in vivo [87].

Metabolic vulnerabilities also offer promising therapeutic opportunities. In CDK4/6 inhibitor-resistant HR+/HER2- breast cancer, mTORC1 hyperactivation induces a metabolic dependency, thereby sensitizing cells to metabolic inhibitors such as metformin and dichloroacetate [88]. Furthermore, natural compounds such as sulforaphane overcome resistance to everolimus in bladder cancer by both sustaining suppression of the AKT-mTOR signaling axis and modulating histone acetylation [45,89,90,91,92].

### 9.3. Synergistic Effects of mTOR Inhibitors with Immunotherapy

The combination of mTOR inhibitors with immune checkpoint blockade is currently under investigation in cancer patients. Preclinical studies have demonstrated that mTOR inhibition upregulates PD-L1 expression on tumor cells. In bladder cancer models, the mTOR inhibitor TAK-228 upregulated cell surface PD-L1, whereas concomitant treatment with an anti-PD-L1 antibody restored T-cell-mediated cytotoxicity [93]. These findings establish a robust mechanistic rationale for this combination strategy. Clinical trials are evaluating such combinations across various malignancies. Notably, a case report documented a profound response in a patient with *PTEN*-mutant poorly differentiated thyroid carcinoma treated with the mTOR inhibitor temsirolimus combined with nivolumab and ipilimumab [94]. However, precise patient stratification is crucial, particularly for malignancies exhibiting Notch pathway activation. This approach facilitates the identification of subpopulations that derive greater clinical benefit from VEGFR/mTOR inhibitors than from immunotherapy alone [95].

## 10. Development of Novel mTOR Allosteric Inhibitors and Bifunctional Molecules

Novel allosteric inhibitors and bifunctional molecules represent promising therapeutic strategies to overcome limitations of conventional mTOR targeting. Bi-steric inhibitors such as RMC-5552 bind both the orthosteric and the allosteric sites of mTORC1, resulting in more profound and more selective inhibition than rapalogs. These compounds have demonstrated preclinical efficacy, particularly when used in combination with KRAS inhibitors [96]. Another innovative approach involves dual-action molecules. For example, YB-3-17 is a bifunctional molecule that simultaneously inhibits mTOR and induces degradation of G1 to S phase transition 1 (GSPT1), demonstrating superior efficacy in glioblastoma cell lines compared to single-agent therapies [97,98].

## 11. Conclusions

The development of resistance to mTOR inhibitors exemplifies the profound adaptability and complexity of cancer biology. Importantly, this resistance is not a singular event but rather a multifaceted phenomenon encompassing the feedback reactivation of parallel pathways, the induction of pro-survival processes such as autophagy, metabolic reprogramming, and the increasingly recognized regulatory layers mediated by epigenetics and non-coding RNAs. Therefore, it is critically important to reconcile diverse—and at times seemingly contradictory—research perspectives, ranging from targeted molecular interrogation to integrative systems biology modeling. A synergistic framework that bridges these distinct scales of inquiry will be essential to advancing the field. The convergence of multi-omics technologies—including genomics, transcriptomics, proteomics, and metabolomics—affords an unprecedented, holistic view of the tumor’s molecular landscape under therapeutic selection pressure. Concurrently, artificial intelligence and machine learning provide the computational power necessary to discern latent patterns, predict resistance evolutionary trajectories, and uncover previously hidden vulnerabilities within this high-dimensional complexity.

## Figures and Tables

**Table 1:** Evidence hierarchy regarding major mechanisms of mTOR inhibitor resistance.

Various Dimensions of mTOR Inhibitor Resistance	Representative Biological Mechanisms	Representative Evidence	Translational Potential	References
Feedback/vertical rewiring	RTK-PI3K-AKT rebound; persistent mTORC2; incomplete 4E-BP1 suppression	Robust preclinical evidence and some clinical rationale	High	[8,9,10,11,12,13]
Autophagy	ULK1 activation; lysosomal recycling	Strong preclinical findings; limited clinical confirmation	Moderate to high	[14,15,16]
Metabolic plasticity	Glycolysis; OXPHOS; lipid and amino acid rewiring	Broad preclinical evidence, but context-dependent	Moderate	[10,17,18,19,20,21,22,23,24,25,26,27,28,29,30,31,32,33,34,35,36,37]
Heterogeneity/TME	Hypoxia; stromal support; immune exclusion	Preclinical studies	Moderate	[24,38,39,40,41,42,43,44,45,46,47]
RNA modifications /ncRNAs	m6A writers/erasers/readers; miRNA/lncRNA/circRNA networks	Exploratory preclinical studies	Low to moderate	[18,48,49,50,51,52,53,54,55,56,57,58,59,60,61,62,63,64,65,66,67,68,69,70,71]
Cuproptosis/other emerging concepts	Mitochondrial stress-linked death programs	Early mechanistic studies	Low to moderate	[72,73,74,75]

## Data Availability

No new data were created or analyzed in this study. Data sharing is not applicable.
